# GRU-ESO Strategy for a Distributed Coil Magnetically Levitated Planar Micromotor

**DOI:** 10.3390/mi15060740

**Published:** 2024-05-31

**Authors:** Chaofan Du, Zhengfeng Ming, Yue Ming, Ding Liu, Yongzheng Li, Yuhu Zhao

**Affiliations:** 1 School of Mechano-Electronic Engineering, Xidian University, Xi’an 710048, China; fyzdcf@yeah.net (C.D.); dliu@xidian.edu.cn (D.L.); 22041212782@stu.xidian.edu.cn (Y.L.); zhaoyuhu@stu.xidian.edu.cn (Y.Z.); 2 State Grid Electric Power Research Institute Nanjing Branch, Nanjing 210000, China; mingyue@sgepri.sgcc.com.cn

**Keywords:** distributed coils, magnetically levitated planar micromotor, GRU, ESO

## Abstract

Traditional magnetic levitation planar micromotors suffer from poor controllability, short travel range, low interference resistance, and low precision. To address these issues, a distributed coil magnetically levitated planar micromotor with a gated recurrent unit (GRU)-extended state observer (ESO) control strategy is proposed in this paper. First, the structural design of the distributed coil magnetically levitated planar micromotor employs a separation of levitation and displacement, reducing system coupling and increasing controllability and displacement range. Then, theoretical analysis and model establishment of the system are conducted based on the designed distributed coil magnetically levitated planar micromotor and its working principles, followed by simulation verification. Finally, based on the established system model, a GRU-ESO controller is designed. An ESO feedback control term is introduced to enhance the system’s anti-interference capability, and the GRU feedforward compensation control term is used to improve the system’s tracking control accuracy. The experimental results demonstrate the reliability of the designed distributed coil magnetic levitation planar micromotor and the effectiveness of the controller.

## 1. Introduction

Magnetically levitated planar micromotors are core components supporting the development of integrated circuits, semiconductors, microfabrication, and other precision manufacturing equipment [1,2]. In recent years, with the rapid development of the precision manufacturing industry, magnetically levitated planar micromotors have become a hot research topic [3]. These devices have advantages such as no friction, no wear, and no noise and can achieve multi-degree-of-freedom precision motion in a vacuum environment [4,5]. Therefore, to meet the requirements of the precision manufacturing field for magnetically levitated planar micromotors, higher requirements have been placed on both mechanical structure design and motion control strategies [6,7].

In the design of mechanical structures, researchers have utilized electromagnetic components and coils to design various platforms, such as magnetically levitated planar micromotors [8,9] and vibration energy harvesters [10], and have achieved significant progress. This paper focuses specifically on magnetically levitated planar micromotors. J W. et al. designed an integrated suspension and displacement magnetically levitated platform [11], in which a Halbach permanent magnet and a three-phase coil jointly generate levitation and horizontal propulsion forces; however, this design has a short travel length and strong coupling. Israeli scholars [12] proposed a separated six-degree-of-freedom precision displacement platform, achieving physical separation of the levitation and displacement mechanisms. The platform consists of three sets of dynamic magnetic structural cantilevers, with each cantilever being in series with a vertical and a horizontal magnetic group, and each magnetic group is composed of two E-type electromagnets. However, it has a small displacement stroke. Kou Baoquan’s team at the Harbin Institute of Technology (HIT) designed a magnetically levitated platform with a dynamic substructure and also adopted the design method of separating levitation and displacement [13]. They used three sets of Y-shaped, interlaced planar motors to achieve horizontal displacement and three sets of gravity compensators for levitation, enabling six-degree-of-freedom displacement of the platform; however, the design of the structure was complicated. Considering the issues of strong coupling, short stroke length, and complex structure in magnetically levitated planar micromotors, this paper designs a distributed coil magnetically levitated planar micromotor based on the separation of levitation and displacement. This design extends the displacement stroke, reduces coupling, and has stronger controllability. In the design process of the distributed coil magnetically levitated planar micromotor, the magnetically levitated system is characterized by high nonlinearity, uncertainty, and susceptibility to disturbances [14]. Therefore, the study of its motion control strategy has become extremely important.

In the design of motion control strategies, it is necessary to propose more advanced control strategies to address the impact on control precision of unknown disturbances in the magnetically levitated planar micromotor and repetitive errors generated during motion. PID is the most typical control strategy [15]. It has the characteristics of a simple control algorithm, strong adaptability, etc., and has been widely used in practical manufacturing. The application of cascade PID control in the controller design for magnetically levitated actuation improves the quality of the system control, but the stability of the system remains poor [16]. On this basis, fuzzy PID was developed for better steady-state accuracy and dynamic responses [17]. Zhou et al. [18] proposed a hybrid fuzzy decoupling control strategy for the leveling and positioning of magnetic levitation wafer carriers. It mainly adopts a primary–secondary structure consisting of a linear primary decoupling control term and a nonlinear secondary intelligent compensation term, aiming to improve the transient stability of the system. In recent years, adaptive robust control has usually been used in the presence of parameter uncertainties and external unknown disturbances in system models [19]. Hu [20] proposed an adaptive robust control strategy for addressing the impact of uncertainty on control accuracy in magnetic levitation platforms, but the design of the control law is complicated. To enhance system stability and robustness, both feedforward and feedback controllers are proposed. In complex excitation environments, issues such as large strokes and uncertain excitation amplitudes in quasi-zero-stiffness isolators can lead to decreased isolation performance and stability. Therefore, a hybrid time-delayed feedforward and feedback controller is proposed to enhance robustness and stability [21]. To enhance the system’s disturbance rejection capabilities, Ma, T. [22] utilized an extended observer to observe external disturbances in the system and provide feedback compensation, thereby achieving improved robustness. At present, the iterative learning control (ILC) strategy is often used as feedforward control to address the impact of repetition errors on control accuracy during reciprocating motion [23]. However, ILC is quite sensitive to uncertain disturbances, parametric uncertainty, and noise, which leads to limitations in industrial applications [24]. Using neural networks to replace iterative learning control is considered. The neural network is equivalent to a black box, predicting tracking errors based on input–output data to approximate the desired trajectory of the control system [25]. To address parameter variations and uncertainties in system models, a neural network learning adaptive robust controller (NNLARC) is adopted, but its prediction accuracy is not high [26]. Therefore, a GRU control strategy [27] is proposed for constructing a GRU neural training network to accurately predict tracking errors. The predicted part is applied as feedforward compensation in the controller, further improving the control accuracy of the control system.

Based on the above analysis, this paper proposes a GRU-ESO control strategy for a designed distributed coil magnetically levitated planar micromotor control system. First, a system model is established based on the dimensions and operating principles of distributed coil magnetically levitated planar micromotors. Then, a GRU-ESO controller is designed based on the established system model. The extended state observer is introduced as a feedback term to suppress unknown disturbances and enhance the disturbance performance of the system. A GRU feedforward control is used to accurately predict the tracking error, and the prediction result is added to the controller as a compensation term to improve the control accuracy of the system. Finally, compared to PID, ILC, and LARC control strategies, the results show that the designed distributed coil magnetically levitated planar micromotor is reliable and the designed controller is effective. In terms of applications in precision manufacturing, further expansion can be achieved.

## 2. Distributed Coil Magnetically Levitated Planar Micromotor and System Modeling

### 2.1. Design of a Distributed Coil Magnetically Levitated Planar Micromotor

**Figure 1:**
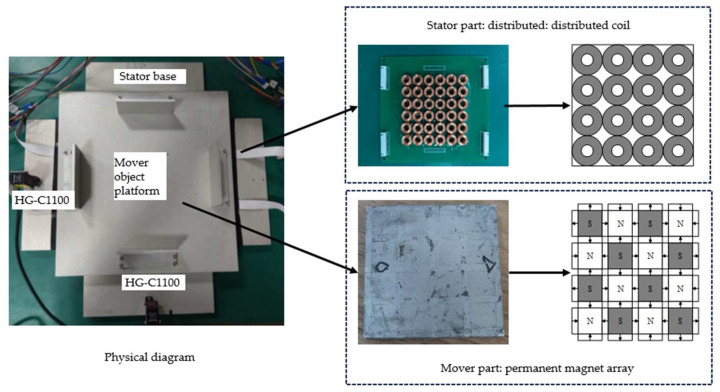
Distributed coil magnetically levitated planar micromotor.

At present, magnetically levitated planar micromotors usually use the design scheme of integrated levitation and displacement [28]. This paper adopts a structure where levitation and displacement are separated. A distributed circular coil array is selected as the stator, and a two-dimensional Halbach permanent magnet array is selected as the mover, reducing system coupling and enhancing controllability. The distributed coil magnetically levitated planar micromotor is shown in Figure 1.

The design process of the stator part is shown in Figure 1: The distributed coil acts as the stator. A single circular coil has the advantages of a simple structure, high replaceability, and expandability. It can extend motion control methods for small-range displacements to larger-range strokes, enhancing the versatility and flexibility of the design [29]. Therefore, in a distributed coil magnetically levitated planar micromotor, the circular coil without an iron core drive is selected as the basic unit of the distributed array, and a single coil is tightly arranged. According to the size of the designed permanent magnet, a 4 × 4 coil array can precisely cover the entire permanent magnet component.

The design process of the mover part is shown in Figure 1. The permanent magnet array acts as the mover, adopting a two-dimensional Helbach permanent magnet array structure [30]. The structure consists of a large and a small combination of two square magnets, where the two-radial magnetization of the magnet arrangement greatly strengthens the magnetic induction intensity of a single side, and a magnetic field of the same volume and the same drive can produce a stronger force. In the figure, N and S are the north and south poles, respectively, and the direction of the arrow is the direction of magnetization.

Based on the above analysis, the parameters of the stator coil and the mover permanent magnet of the designed distributed coil magnetically levitated planar micromotor are shown in Table 1 and Table 2.

### 2.2. System Modeling

This section will establish the dynamic model of the distributed coil magnetically levitated planar micromotor system. Section 2.1 describes the design of the mechanical structure of the magnetic levitation planar micromotor, including the distributed coil array as the stator and the permanent magnet array as the mover.

#### 2.2.1. Analysis of the Magnetic Field Density

The permanent magnet array in this paper contains two types of square magnets, differing only in size; therefore, the magnetic induction intensity calculation method for square magnets is used in the analysis. The expression for the magnetic induction intensity of a single rectangular magnet is as follows:

(1) $$\{\begin{array}{ll} B_{x} & =-\frac{\mu_{0}I_{M}}{8\pi h}[\psi (a-x,y,z)+\psi (a-x,b-y,z)-\psi (x,y,z)-\psi (x,b-y,z)]{|}_{0}^{h}\\ B_{y} & =-\frac{\mu_{0}I_{M}}{8\pi h}[\psi (b-y,x,z)+\psi (b-y,a-x,z)-\psi (y,x,z)-\psi (y,a-x,z)]{|}_{0}^{h}\\ B_{z} & =-\frac{\mu_{0}I_{M}}{4\pi h}[\Phi (y,a-x,z)+\Phi (b-y,a-x,z)+\Phi (x,b-y,z)+\Phi (b-y,x,z)\\ & +\;\Phi (a-x,b-y,z)+\Phi (y,x,z)+\Phi (a-x,y,z)+\Phi (x,y,z)]{|}_{0}^{h} \end{array},$$

where a, b, and h are the length, width, and height of the magnet, respectively; any point outside the magnetic field is P(x,y,z). Moreover,

(2) $$\psi (\alpha_{1},\alpha_{2},\alpha_{3})=\ln\frac{\sqrt{{\alpha_{1}}^{2}+{\alpha_{2}}^{2}+{\left(\alpha_{3}-z_{0}\right)}^{2}}-\alpha_{2}}{\sqrt{{\alpha_{1}}^{2}+{\alpha_{2}}^{2}+{\left(\alpha_{3}-z_{0}\right)}^{2}}+\alpha_{2}},$$

(3) $$\Phi (\beta_{1},\beta_{2},\beta_{3})=\{\begin{array}{cc} \arctan\left[\frac{\beta_{1}}{\beta_{2}}\frac{\beta_{3}-z_{0}}{\sqrt{{\beta_{1}}^{2}+{\beta_{2}}^{2}+{\left(\beta_{3}-z_{0}\right)}^{2}}}\right] & \left(y\neq 0\right)\\ 0 & \left(y=0\right) \end{array},$$

According to the definition of the surface current density $J=I_{M}/h$, to determine the surface current density, let us derive the expression for J. Assuming the coordinates of the point p(a/2,b/2,h), the $B_{z}$ of (1) can be obtained:

(4) $$J=-\frac{B_{z}\pi }{\mu_{0}\left[\Phi (\frac{a}{2},\frac{b}{2},h)+\Phi (\frac{b}{2},\frac{a}{2},h)\right]{|}_{0}^{h}},$$

This paper utilizes Ansys Maxwell finite element software for magnetic field simulation. When p(5,5,5) and $B_{z1}=0.4249T$, substituting the value of $B_{z1}$ into (4), the equivalent surface current density of the rectangular large magnet is determined to be $J_{1}=1.0144\times 10^{6}{A/m}^{2}$. Additionally, when p(5,2.5,2.5) and $B_{z2}=0.3533T$, the equivalent surface current density of the rectangular small magnet is determined to be $J_{2}=9.9681\times 10^{5}{A/m}^{2}$.

Therefore, substituting the surface current density J into (1), it can be observed that the magnetic induction intensity of a single rectangular magnet at any point outside the magnet is related only to the coordinates of the point of calculation and the surface current density of the magnet, which are obtained as follows:

(5) $$(B_{x},B_{y},B_{z})=f(x,y,z,J),$$

Due to the arrangement of the Halbach permanent magnet array, the magnetic field generated at any point Q(x,y,z) outside the Halbach permanent magnet array can be calculated using the magnetic induction intensity components in three directions of a single magnetic steel and the superposition principle, which are as follows:

(6) $$\{\begin{array}{c} B_{Qx}={B_{zx}}^{\prime }-{B_{-zx}}^{\prime }+{B_{xz}}^{\prime }-{B_{-xz}}^{\prime }-{B_{yx}}^{\prime }-{B_{-yx}}^{\prime }\\ B_{\mathrm{Qy}}={B_{zy}}^{\prime }+{B_{-zy}}^{\prime }+{B_{xx}}^{\prime }+{B_{-xx}}^{\prime }+{B_{yz}}^{\prime }-{B_{-yz}}^{\prime }\\ B_{\mathrm{Qz}}={B_{zz}}^{\prime }-{B_{-zz}}^{\prime }+{B_{xy}}^{\prime }-{B_{-xy}}^{\prime }+{B_{yy}}^{\prime }-{B_{-yy}}^{\prime } \end{array},$$

where ${B_{xx}}^{\prime }$, ${B_{xy}}^{\prime }$, and ${B_{xz}}^{\prime }$ are the magnetic induction intensities in the x, y, and z directions, respectively, with the magnetization direction along the positive *x*-axis; ${B_{-xx}}^{\prime }$, ${B_{-xy}}^{\prime }$, and ${B_{-xz}}^{\prime }$ are the magnetic induction intensities in the x, y, and z directions, respectively, with the magnetization direction along the negative *x*-axis; ${B_{yx}}^{\prime }$, ${B_{yy}}^{\prime }$, and ${B_{yz}}^{\prime }$ are the magnetic induction intensities in the x, y, and z directions, respectively, with the magnetization direction along the positive *y*-axis; ${B_{-yx}}^{\prime }$, ${B_{-yy}}^{\prime }$, and ${B_{-yz}}^{\prime }$ are the magnetic induction intensities in the x, y and z directions, respectively, with the magnetization direction along the negative *y*-axis; ${B_{zx}}^{\prime }$, ${B_{zy}}^{\prime }$, and ${B_{zz}}^{\prime }$ and ${B_{yx}}^{\prime }$, ${B_{yy}}^{\prime }$, and ${B_{yz}}^{\prime }$ are the magnetic induction intensities in the x, y, and z directions, respectively, with the magnetization direction along the positive *z*-axis; and ${B_{-zx}}^{\prime }$, ${B_{-zy}}^{\prime }$, and ${B_{-zz}}^{\prime }$ are the magnetic induction intensities in the x, y, and z directions, respectively, with the magnetization direction along the negative *z*-axis. The specific expressions can be found in Appendix A.

The unknown quantity in (6) is only the calculated point Q and the surface current density $J_{1}$, $J_{2}$ of the magnetic steel. Therefore, the magnetic induction intensity outside the Halbach permanent magnet array is expressed as follows:

(7) $$B_{Q}=(B_{Qx},B_{\mathrm{Qy}},B_{\mathrm{Qz}})=F(x,y,z,J_{1},J_{2}),$$

**Figure 2:**
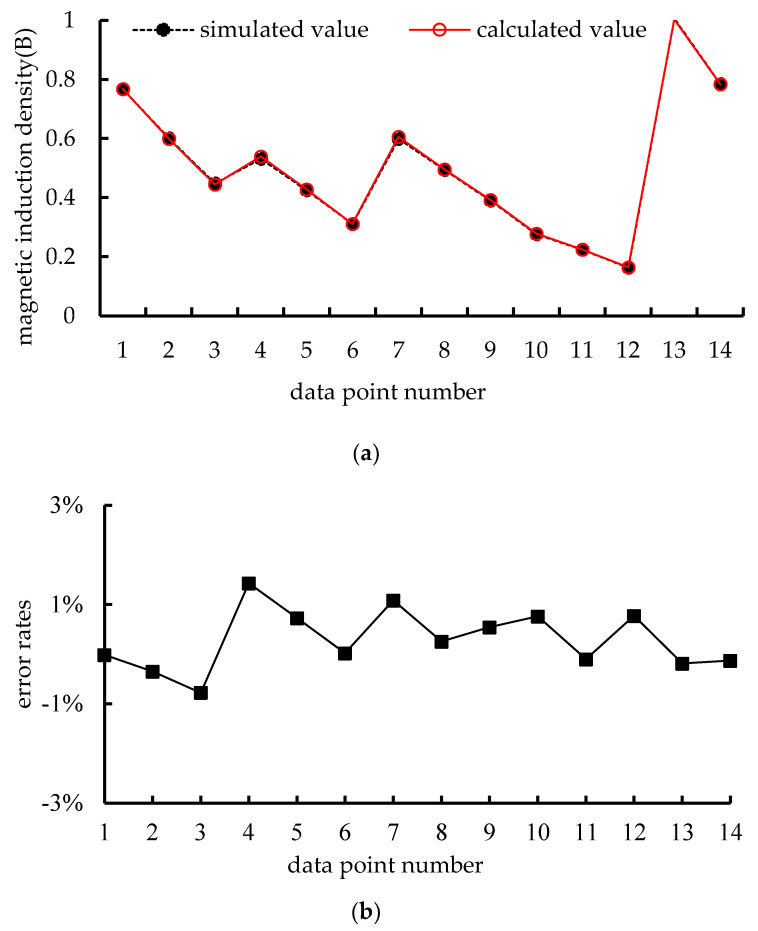
Results of magnetic field density: (**a**) comparison results between calculation and simulation, (**b**) error rates.

To verify the correctness of (7), the calculated results based on the above formula and simulated results obtained using Ansys Maxwell finite element are compared. The results of magnetic induction density are shown in Figure 2.

The comparison results between calculation and simulation in Figure 2a and the error rates in Figure 2b show that the magnetic induction density obtained from the calculation and the simulation of (7), the results are basically the same, and the error rates are all between −1% and 1%. This shows the correctness of the above (7). Next, based on the magnetic induction intensity, the electromagnetic force is calculated.

#### 2.2.2. Analysis of the Electromagnetic Force

According to the working principle of the distributed coil magnetically levitated planar micromotor, the coil in the magnetic field will experience ampere force when a current passes through it. The force on a current-carrying circular coil in a magnetic field can be calculated using closed-path integrals. However, using integrals for magnetic field calculations, as in (7), can make the process overly complex. Therefore, this paper adopts the approach of considering a circular coil as a superposition of finite current loops. First, each current loop is divided into several current line elements. Then, the force on each line element is decomposed into three components using the approach in (7). Finally, summing these components yields the total force on a single coil in the magnetic field.

**Figure 3:**
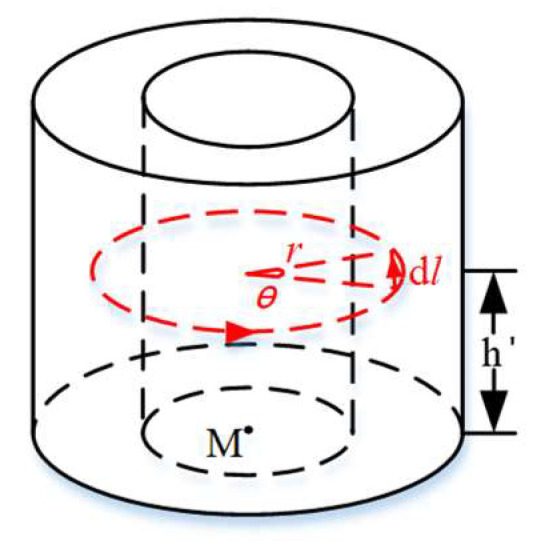
Schematic diagram of the coil current loop.

Assuming that the center of the bottom of a coil is located at the coordinate M(x,y,z) within the Halbach permanent magnet array and that each layer of current loops is counted from the innermost to the outermost coil with i(i=0~3) and from the bottom to the top layer with j(j=0~3), then one current loop can be determined. The (i,j)-th current loop is taken for analysis, with a radius of r, a height of $h^{\prime }$, and a center angle of θ, as shown in Figure 3.

Assuming a current loop divided into *m* line elements, when the line elements are short enough, the *n*-th (*n* = 0~*m* − 1) line element in the Halbach permanent magnet array coordinates is $\left(x^{\prime },y^{\prime },z^{\prime }\right)$ and is expressed as follows:

(8) $$\{\begin{array}{l} x^{\prime }=x-r\sin(n\theta )\\ y^{\prime }=y+r\cos(n\theta )\\ z^{\prime }=z+h^{\prime } \end{array},$$

(9) $$r=\frac{D_{1}+d}{2}+i\cdot d,$$

(10) $$h^{\prime }=\frac{d}{2}+j\cdot d,$$

(11) $$\theta =\frac{2\pi }{m},$$

According to the Lorentz force F = BIL, the force on the line element can be obtained:

(12) $$\{\begin{array}{l} F_{x^{\prime }}=-B_{z^{\prime }}I(r\cdot \theta )\sin(n\theta )\\ F_{y^{\prime }}=B_{z^{\prime }}I(r\cdot \theta )\cos(n\theta )\\ F_{z^{\prime }}=B_{x^{\prime }}I(r\cdot \theta )\sin(n\theta )-B_{y^{\prime }}I(r\cdot \theta )\cos(n\theta ) \end{array},$$

According to (17), the force on a single coil can be expressed as:

(13) $$\begin{array}{ll} F & =\sum_{i=0}^{n_{D}}\sum_{j=0}^{n_{h}}\sum_{n=0}^{m-1}\left(F_{x}^{'},F_{y}^{'},F_{z}^{'}\right)=G(x,y,z,I)\\ & =\sum_{i=0}^{n_{D}}\sum_{j=0}^{n_{h}}\sum_{n=0}^{m-1}g(x,y,z,i,j,n,I,m) \end{array},$$

**Figure 4:**
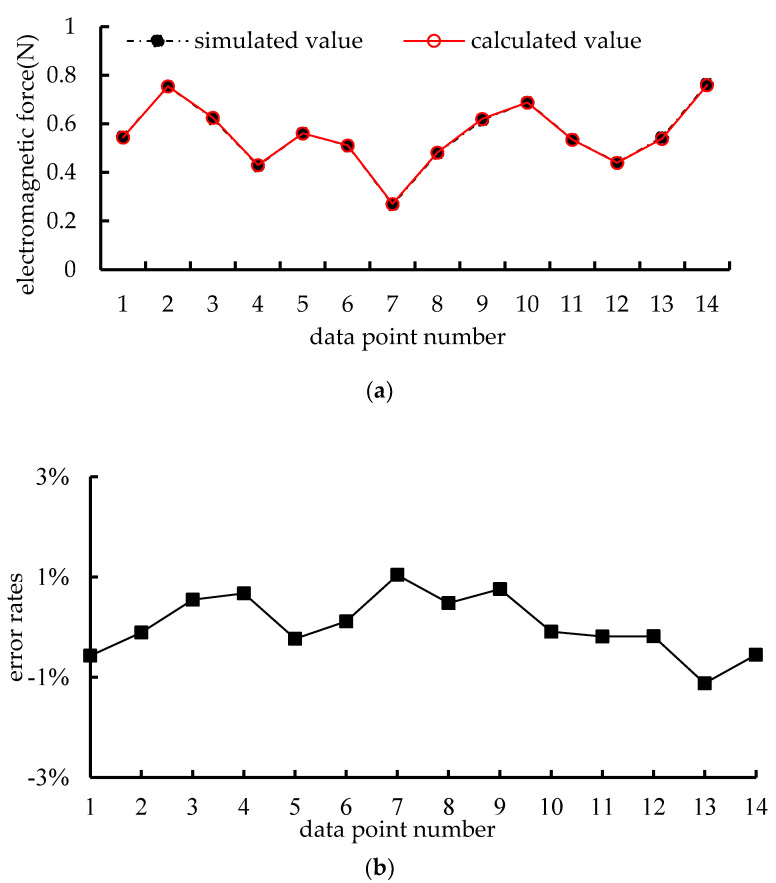
Results of electromagnetic force: (**a**) comparison results between calculation and simulation, (**b**) error rates.

To verify the correctness of (13), the calculated results based on the above formula and simulated results obtained using Ansys Maxwell finite element are compared. The results of electromagnetic force are shown in Figure 4.

The comparison results between calculation and simulation in Figure 4a and the error rates in Figure 4b show that the electromagnetic forces obtained from the calculation and the simulation of (13), the results are basically the same, and the error rates are all between −1% and 1%. This shows the correctness of the above (13). Next, based on the coordinate relationship between the two, a system model of a distributed coil magnetically levitated planar micromotor is established.

#### 2.2.3. Analysis of the System Model

**Figure 5:**
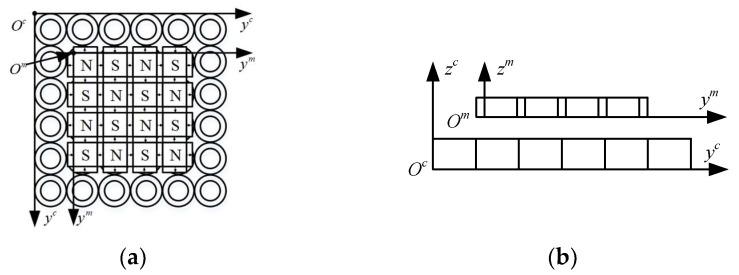
Schematic diagram of the global and local coordinate system: (**a**) top view, (**b**) main view.

According to the electromagnetic force analysis of the distributed coil magnetically levitated planar micromotor, it is necessary to unify the coordinates into the same coordinate system to calculate the electromagnetic force. Define the global coordinate system $\left(x^{c},y^{c},z^{c}\right)$ and the local coordinate system $\left(x^{m},y^{m},z^{m}\right)$. The schematic diagram is shown in Figure 5.

During the motion of a distributed coil magnetically levitated planar micromotor, suppose the coordinates of a certain coil in the global coordinate system are represented as $p^{c}(x_{p}^{c},y_{p}^{c},z_{p}^{c})$ and that those in the local coordinate system are represented as $p^{m}(x_{p}^{m},y_{p}^{m},z_{p}^{m})$. According to positional kinematics [28], there is a relationship between $p^{c}$ and $p^{m}$:

(14) [xpcypczpc1]=Tmc[xpmypmzpm1],

where Tmc is the transformation matrix of the local coordinate system to the global coordinate system and Tcm=Tm−1c.

Therefore, if the coordinates $p^{c}$ of a coil are measured in the global coordinate system, the coordinates $p^{m}$ of the coil in the local coordinate system can be obtained:

(15) [xpmypmzpm1]=Tcm[xpcypczpc1],

Substituting $p^{m}$ into (13) obtains the force on a single coil. Because the force on the coil is linearly related to the input current, the driving force of a single coil of the planar micromotor can be expressed as follows:

(16) $$F_{n}=(F_{nx},F_{ny},F_{nz})=-I_{n}G(x_{p}^{m},y_{p}^{m},z_{p}^{m},1)=K_{Fn}I_{n},$$

where $K_{Fn}$ is the defined mapping vector from the current to the driving force, which is equal to the driving force, when a current of 1 A is passed through a single coil at a certain position, depending only on the position of the coil in the local coordinate system.

For the planar micromotor, in addition to the driving forces from the driving coils in the *x*, *y*, and *z* directions, it also experiences torques in three directions. The coordinates of the center of mass of the permanent magnet array used in this paper in the local coordinate system is $C^{m}(x_{C}^{m},y_{C}^{m},z_{C}^{m})$, the coil local coordinate is $p^{m}(x_{p}^{m},y_{p}^{m},z_{p}^{m})$, and the coil height is $h_{c}$. The torques in the three directions are expressed as follows:

(17) $$\{\begin{array}{l} T_{x}=F_{z}\cdot (y_{C}^{m}-y_{p}^{m})-F_{y}\cdot [z_{C}^{m}-(z_{p}^{m}+\frac{h_{c}}{2})]\\ T_{y}=F_{z}\cdot (x_{p}^{m}-x_{C}^{m})+F_{x}\cdot [z_{C}^{m}-(z_{p}^{m}+\frac{h_{c}}{2})]\\ T_{z}=F_{x}\cdot (y_{p}^{m}-y_{C}^{m})+F_{y}\cdot (x_{C}^{m}-x_{p}^{m}) \end{array},$$

Since the designed permanent magnet array and coil dimensions are fixed, the center of mass coordinates of the permanent magnet array and the coil height are constant. Therefore, the torques for a single coil (17) can be expressed as follows:

(18) $$T_{n}=(T_{nx},T_{ny},T_{nz})=K_{Mn}F_{n}=K_{Mn}K_{Fn}I_{n},$$

where $K_{Mn}$ is the defined mapping vector from the drive to the torque, and it only depends on the position of the coil in the local coordinate system.

Based on the driving forces (16) and torques (18) of a single drive coil on a planar micromotor, the effect of a single drive coil on a planar micromotor is regarded as a drive vector, expressed as follows:

(19) $$S_{n}={\left[{\begin{array}{cc} F_{n} & T \end{array}}_{n}\right]}^{T}=[{\begin{array}{cccccc} F_{nx} & F_{ny} & F_{nz} & T_{nx} & T_{ny} & T_{nz}] \end{array}}^{T}=K_{n}I_{n},$$

where $K_{n}$ is the defined mapping vector from the current to the driving, and it only depends on the position of the coil in the local coordinate system.

The Halbach permanent magnet array designed in this paper is coordinated with the dimensions of the coils. When the permanent magnet array is at any position within the coil array, it can be covered by 4 × 4 = 16 coils. Therefore, at any time, the planar micromotor is subjected to the drive vector from 16 coils, expressed as follows:

(20) $$S=S_{1}+S_{2}+\ldots +S_{15}+S_{16}=[\begin{array}{ccc} K_{1} & \ldots & K_{16}] \end{array}[{\begin{array}{ccc} I_{1} & \ldots & I_{16}] \end{array}}^{T}=KI,$$

According to (16), (18), and (20), it can be expressed as follows:

(21) $$K={\left[\begin{array}{cccc} F_{1x} & F_{2x} & \ldots & F_{16x}\\ F_{1y} & F_{2y} & \ldots & F_{16y}\\ F_{1z} & F_{2z} & \ldots & F_{16z}\\ T_{1x} & T_{2x} & \ldots & T_{16x}\\ T_{1y} & T_{2y} & \ldots & T_{16y}\\ T_{1z} & T_{2z} & \ldots & T_{16z} \end{array}\right]|}_{I={\left[\begin{array}{ccc} 1 & \ldots & 1 \end{array}\right]}^{T}},$$

where K is the matrix consisting of the driving forces and torques when 16 working coils are supplied with a current of 1 A.

According to the concept of separating the suspension mechanism from the displacement mechanism and the analysis above, the planar micromotor will experience a total driving vector S at any time:

(22) $$S={\left[\begin{array}{cccccc} F_{x} & F_{y} & F_{z} & T_{x} & T_{y} & T_{z} \end{array}\right]}^{T},$$

Then, according to Newton’s second law and the law of torque rotation, the dynamic model of the planar micromotor can be obtained as follows:

(23) $$S=\left[\begin{array}{c} F_{x}\\ F_{y}\\ F_{z}\\ T_{x}\\ T_{y}\\ T_{z} \end{array}\right]=\left[\begin{array}{cccccc} m & & & & & \\ & m & & & & \\ & & m & & & \\ & & & J_{x} & & \\ & & & & J_{y} & \\ & & & & & J_{z} \end{array}\right]\left[\begin{array}{c} a_{x}\\ a_{y}\\ a_{z}\\ \alpha_{x}\\ \alpha_{y}\\ \alpha_{z} \end{array}\right]+f,$$

where $F_{x}$, $F_{y}$, and $F_{z}$ are the total driving force components generated by the 16 coils, respectively; $T_{x}$, $T_{y}$, and $T_{z}$ are the total torque components generated by the 16 coils, respectively; m is the mass of the mover of planar micromotor; $J_{x}$, $J_{y}$, and $J_{z}$ are the rotational torques of inertia of the mover around the three axes of *x*, *y,* and *z*, respectively; $a_{x}$, $a_{y}$, and $a_{z}$ are the accelerations along the three directions of *x*, *y,* and *z*, respectively; $\alpha_{x}$, $\alpha_{y}$, and $\alpha_{z}$ are the angular accelerations around the three axes of *x*, *y,* and *z,* respectively; and f is the total disturbance vector during the motion of the planar micromotor.

**Figure 6:**
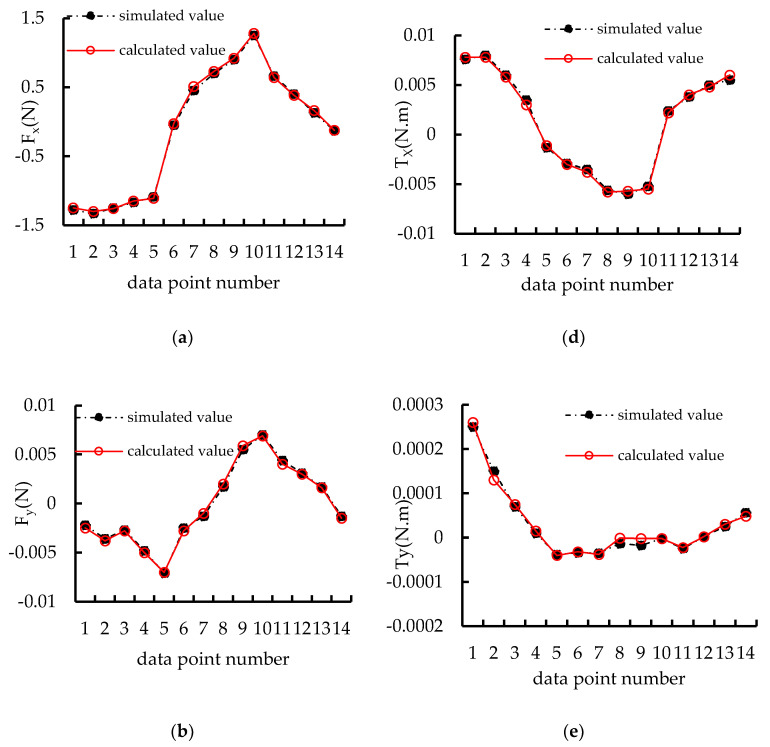

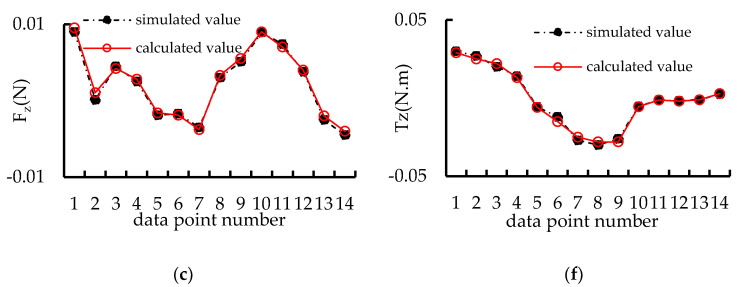
Result of electromagnetic forces and torques: (**a**) comparison results between calculation and simulation of $F_{x}$; (**b**) comparison results between calculation and simulation of $F_{y}$; (**c**) comparison results between calculation and simulation of $F_{z}$; (**d**) comparison results between calculation and simulation of $T_{x}$; (**e**) comparison results between calculation and simulation of $T_{y}$; (**f**) comparison results between calculation and simulation of $T_{z}$.

To verify the correctness of (23), the calculated results based on the above formula and simulated results obtained using Ansys Maxwell finite element are compared. The results of electromagnetic forces and torques are shown in Figure 6.

In Figure 6, it can be observed that the simulated values of the electromagnetic force and torque are consistent with the trend of the results of calculation, indicating the correctness of establishing the model (23).

Finally, the dynamics model of the distributed coil magnetically levitated planar micromotor (23), a second-order undamped system can be expressed as:

(24) $$\theta \ddot{x}=u+f.$$

where x and $\ddot{x}$ are the position and acceleration of the system’s mover, respectively; θ is the system model parameter, i.e., the mass of the mover; and u is the control input, i.e., the combined force or torque in each axis.

## 3. Design of the Controller

To address the susceptibility of the distributed coil magnetically levitated planar micromotor to uncertain disturbances, ESO is used to suppress disturbances and enhance robustness, which inherently introduces a certain degree of conservatism to the accuracy of the practical control. Therefore, before executing motion tasks, a GRU neural network is used to predict the ESO tracking trajectory. The predicted tracking error serves as a feedforward compensation term and together with the ESO feedback compensation term, it constitutes the whole GRU-ESO system strategy.

### 3.1. Design of ESO

For the distributed coil magnetically levitated planar micromotor, uncertain factors such as unknown load parameters on the mover and external unknown disturbances can reduce the control tracking accuracy of the system and affect its stability. Therefore, the introduction of the ESO enhances the system’s robustness. According to (24), the dynamic formula of the planar micromotor can be obtained as follows:

(25) $$\left\{ \begin{array}{l} \dot{x}=v\\ \dot{v}=\frac{1}{\theta }u+f(t)=\ddot{x} \end{array}\right.,$$

where f(t) is the set total effect of uncertain nonlinear disturbances.

Set f(t) in (25) to $x_{3}$; then, $x_{1}=x$ and $x_{2}=v$. Additionally, ${\dot{x}}_{3}=\omega (t)$ is obtained as follows:

(26) $$\left\{ \begin{array}{l} {\dot{x}}_{1}=x_{2}\\ {\dot{x}}_{2}=f(t)+bu\\ y=x_{1} \end{array}\right.,$$

(27) $$\left\{ \begin{array}{l} {\dot{x}}_{1}=x_{2}\\ {\dot{x}}_{2}=x_{3}+bu\\ {\dot{x}}_{3}=\omega (t)\\ y=x_{1} \end{array}\right.$$

Letting $z_{i}$ be considered as an observed value of the system state $x_{i}$(*i =* 1, 2, 3 ....) and the observation error be $z_{i}-x_{i}$, the final expression for the nonlinear ESO can be obtained as follows:

(28) $$\left\{ \begin{array}{l} e=z_{1}-y\\ {\dot{z}}_{1}=z_{2}-\beta_{1}e\\ {\dot{z}}_{2}=z_{3}-\beta_{2}fal(e,\alpha_{1},\varepsilon )+bu\\ {\dot{z}}_{3}=-\beta_{3}fal(e,\alpha_{2},\varepsilon ) \end{array}\right.,$$

where e is the system error; u, y, and b are the control volume of the system, the output, and the coefficients, respectively; $\beta_{1}$, $\beta_{2}$, and $\beta_{3}$ are the output error correction gains; $\alpha_{1}$ and $\alpha_{2}$ are nonlinear factors, generally taken as 0.5, 0.25, and 0.125; ε is the filtering factor, with ε>0; and $z_{1}$, $z_{2}$, and $z_{3}$ are the tracking signal of y, the differential signal of $z_{1}$, and the tracking signal of the system disturbance, respectively.

ESO uses a nonlinear expression to compensate for losses due to disturbances in the system, to reduce the magnitude of error variations, and to improve the control performance. Its expression is as follows:

(29) $$fal(e,\alpha ,\varepsilon )=\left\{ \begin{array}{l} \frac{e}{\varepsilon^{1-\alpha }}\begin{array}{ccccc} & & & & \begin{array}{cc} & \left|e\right|\leq \varepsilon \end{array} \end{array}\\ {\left|e\right|}^{\alpha }sign(e)\begin{array}{ccc} & & \left|e\right|>\varepsilon \end{array} \end{array}\right.,$$

where ε>0, 0<α<1, with ε and α being constants.

According to the literature [31], the system is asymptotically stable about the zero equilibrium point at ω(t)=0 if the gain coefficients of the ESO are $\beta_{1}>0$, $\beta_{2}>0$, $\beta_{2}>0$, and $\beta_{1}\beta_{2}>\beta_{3}$. Therefore, the following state observer can be realized:

(30) $$\left\{ \begin{array}{l} z_{1}(t)\to x_{1}(t)\\ z_{2}(t)\to x_{2}(t)\\ z_{3}(t)\to x_{3}(t)=f(x_{1},x_{2},t) \end{array}\right..$$

ESO is robust to unknown external disturbances but fails to fully compensate for the errors existing in the system model. Next, to improve the control tracking accuracy of the system, a GRU neural network is introduced to predict the residual error.

### 3.2. Design of GRU Control

A gated recurrent unit (GRU) is a derivation and improvement of a recurrent neural network (RNN). Compared to RNN [32], firstly, the implementation of a GRU is more straightforward, and the GRU is easier to understand and implement. Secondly, the GRU can better handle long-term dependencies and the vanishing gradient problem, thereby improving the performance of the model and enhancing the control accuracy of the system. Thirdly, the GRU can be computed in parallel, accelerating the model training process, thus reducing the time required and enabling faster attainment of the desired position. Compared to long short-term memory (LSTM) [33], firstly, the GRU has fewer parameters and is easier to converge. In this experiment’s GRU-ESO controller design, the ESO control part also has several parameters. To reduce the number of parameters, the GRU is chosen. Secondly, the GRU has only two gates, while LSTM has three gates, making the GRU’s computation time relatively shorter. A shorter computation time allows the motion trajectory of the experimental platform to reach the desired position more quickly. Third, with a small amount of collected data, the GRU exhibits better predictive performance. Due to limited equipment resources in the laboratory used, this study did not collect a large amount of data.

**Figure 7:**
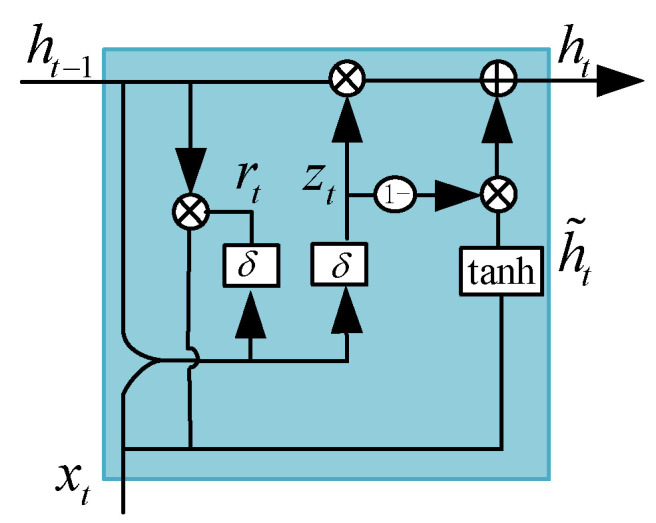
Structure of a unit (GRU).

In conclusion, the experimental research in this paper shows that the GRU converges quickly with short computation times, reaching the desired position rapidly. The data collection volume is not large, and the precision of the control is high. Therefore, it is more suitable for a distributed coil magnetically levitated planar micromotor. The structure of the GRU mainly consists of an updater gate and a reset gate, as shown in Figure 7. The update gate controls the degree to which information from the previous hidden state is retained in the current hidden state, and the reset gate controls the degree to which information from the previous hidden state is forgotten in the current hidden state. Through these two gate mechanisms, the GRU can effectively select and update information in the hidden state, thus modeling sequential data.

First, according to the splicing matrix of the current time $x_{t}$ and the hidden state $h_{t-1}$ in the previous time, we obtain the reset gate’s gating state $r_{t}$ and the update gate’s gating state $z_{t}$ at time *t*:

(31) $$\left\{ \begin{array}{l} r_{t}=\delta (\left[W_{r}x_{t}\right]+\left[U_{r}h_{t-1}\right])\\ z_{t}=\delta (\left[W_{z}x_{t}\right]+\left[U_{z}h_{t-1}\right]) \end{array}\right.,$$

where δ denotes the logistic sigmoid function; $W_{r}$, $U_{r}$ and $W_{Z}$, $U_{Z}$ are weight matrices that are learned; and $W_{Z}\in R_{n\times m}$ and $U_{Z}\in R_{n\times m}$.

Then, the reset gate $h_{t-1}$ determines the degree of retention of the information from the previous time. $h_{t-1}$ is computed after reset gate processing with $x_{t}$ to obtain the candidate hidden layer state ${\tilde{h}}_{t}$ at time *t*:

(32) $${\tilde{h}}_{t}=\tanh(\left[Wx_{t}\right]+\left[U(r_{t}⊙h_{t-1})\right]),$$

where W and U are weight matrices and $W\in R_{n\times m}$ and $U\in R_{n\times m}$ are Hadamard products.

Finally, according to $z_{t}$ and ${\tilde{h}}_{t}$, the current moment information is processed and combined with $h_{t-1}$, the historical moment information, to calculate the output state of the GRU at the current time:

(33) $$h_{t}=z_{t}h_{t-1}+(1-z_{t}){\tilde{h}}_{t},$$

The GRU can efficiently capture key information in long-term sequences. At each time step, the GRU dynamically retains and updates the relevant information, transmitting it to the next time step of the network until the model processes the complete sequence. The hidden state $h_{t}$ of the last time step is passed as input to the fully connected layer and generates the final prediction result through linear mapping.

### 3.3. Design of GRU-ESO Controller

To further improve the tracking control accuracy of the distributed coil magnetically levitated planar micromotor, the introduction of a GRU neural network is considered to predict the residual tracking error of ESO, thus improving the tracking performance. This paper utilizes a GRU network to predict the tracking error of the ESO for a maglev planar motor, forming the GRU-ESO control strategy.

It is assumed that the reference of one-axial motion is

(34) $$P={\left(p_{T},p_{2T},p_{T},\cdot \cdot \cdot ,p_{nT}\right)}^{T},$$

where T is the sampling time, n is the number of sampling points, and p represents the sequence of the reference positions. Through differentiating, the velocity, acceleration, and jerk of the reference can be obtained as follows:

(35) $$\begin{array}{l} v=(v_{T},v_{T},\cdot \cdot \cdot ,v_{nT}{)}^{T}\\ a=(a_{T},a_{T},\cdot \cdot \cdot ,a_{nT}{)}^{T}\\ j=(j_{T},j_{T},\cdot \cdot \cdot ,j_{nT}{)}^{T} \end{array},$$

Based on the structural unit model of the GRU network for learning and prediction, as well as the distributed coil magnetically levitated planar micromotor, the design of the input for the training set at time *t* is

(36) $$x_{t}={\left(p_{t},p_{t-T},p_{t+T},v_{t},v_{t-T},v_{t+T},a_{t},a_{t-T},a_{t+T},j_{t},j_{t-T},j_{t+T}\right)}^{T}.$$

This is a 12-dimensional vector. The output of the training set is the tracking error filtered by a low-pass zero-phase filter.

**Figure 8:**
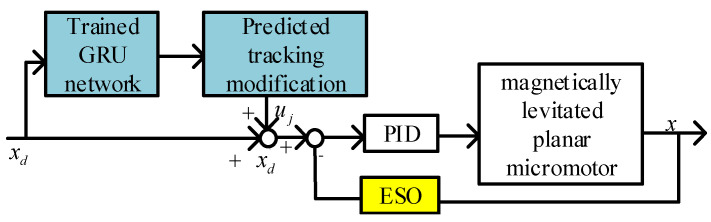
Structure of GRU-ESO control.

First, by using the GRU network to learn about the distributed coil magnetically levitated planar micromotor, the internal characteristics of the platform structure are fully acquired. Then, based on the input data, the output position is obtained to predict the error at the current time *t.* Finally, by compensating for the predicted error, it is combined with the ESO controller to form the final GRU-ESO strategy, as shown in Figure 8.

## 4. Experimental Results and Discussion

### 4.1. Setup and Control of Experiments

**Figure 9:**
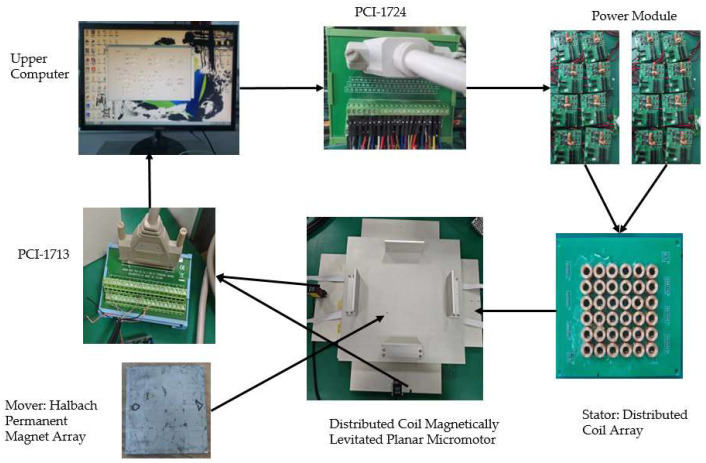
Experimental diagram of a distributed coil magnetically levitated planar micromotor.

The proposed GRU-ESO strategy in this paper is validated on the distributed coil magnetically levitated planar micromotor in the laboratory, as shown in Figure 9. The mass of the mover is approximately 3.67 kg. The control system consists of the PCI-1724 and PCI-1713 boards, a real-time operating system, an HG-C1100 miniature laser position sensor, and VB software. Both the PCI-1724 and PCI-1713 boards are analog output cards with a voltage output range of −10 to 10 V. The PCI-1713 has a total of 32 analog input channels with a resolution of 12 bits. The PCI-1724 has 32 analog output channels with a resolution of 14 bits. The output signal of the sensor is linearly related to the measured distance, providing a voltage analog signal ranging from 0 to 5 V. When the sensor reads the position information of the mover, the output signal is transmitted to the host computer through the PCI-1713. Then, the host computer executes the controller algorithm at a sampling frequency of *fs* = 5 kHz, where the sampling frequency is determined by the hardware settings and complexity of the algorithm. Finally, the calculated results are sent down to the powered power module via the PCI-1724. After processing, the power module sends the required current to the designed distributed coil. The controller executes the algorithm with a sampling frequency of *fs* = 5 kHz. Different desired trajectories in the single *x*-axis are used for validation. The following indicators are used to evaluate the quality of the control strategy, i.e.,

- $e_{RMS}=(\frac{1}{T}\int_{0}^{T}|e_{t}{|}^{2}dt{)}^{1/2}$ is the root-mean-square (RMS) value of the tracking error, where *T* is the total time;
- $e_{M}=\underset{t}{\max}\left\{\left|e(t)\right|\right\}$ is the maximal (M) absolute value of the tracking error over the total time.

To provide a sufficient comparison, a traditional PID, ILC, PID-ESO, and the proposed GRU-ESO are implemented.

C1: PID controller—A common parallel PID form is used, and the transfer function is

(37) $$C(s)=K+K_{i}/s+K_{d}s.$$

The PID parameters are set as $K_{P}=100$, $K_{i}=10$, and $K_{d}=1$. All these parameters guarantee the stability of the overall controller.

C2: ILC—Iterative learning control. ILC is used as a feedback control. The control law is

(38) $$u_{j+1}=Q[u_{j}+e_{j}].$$

where $u_{j}$ and $e_{j}$ are the input controls of the trajectory and the tracking error of the trajectory, respectively; j is number of iterations. *Q* is a first-order low-pass filter, usually taking the value 1.

C3: PID-ESO—Extended State Observer. ESO is very robust. Its parameters are as follows: $\beta_{1}=0.01$, $\beta_{2}=200$, $\beta_{3}=1000$; $\alpha_{1}=0.5$, $\alpha_{2}=0.25$; ε=0.01.

C4: GRU-ESO—The proposed control strategy is shown in Figure 8 in this paper. The parameters of the adaptive and robust terms are the same as those in C3.

To validate the tracking performance of the proposed control strategy in different situations, the following test sets are used:

1. Set 1: To verify the nominal tracking performance, the experiment is run without disturbances, i.e., f(t)=0 and payload.
2. Set 2: To verify the robustness to unknown external disturbances, random external and internal disturbances were added to the control input.
3. Set 3: To verify the robustness to unknown external disturbances and parameter uncertainties, based on Set 2, a 1 kg payload is added on the mover.

**Figure 10:**
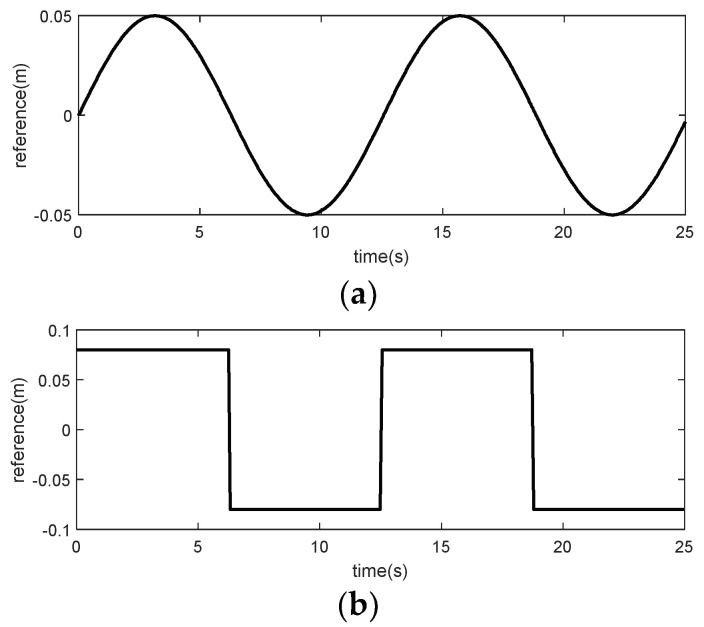
Reference of *y*_1_ and *y*_2_ in the control validation: (**a**) reference in *y*_1_: sine curve; (**b**) reference in *y*_2_: square wave curve.

**Figure 11:**
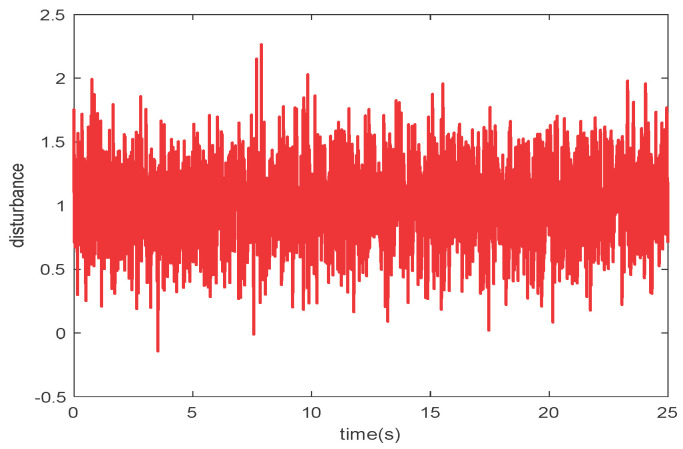
Disturbances in control validation.

To validate the effectiveness and correctness of the proposed control strategy, two different kinds of reference trajectories shown in Figure 10 and disturbances shown in Figure 11 are used. The first is a sine curve, $y_{1}=0.05\sin(0.5t)$; the second is a square wave curve, $y_{2}=0.08\sin(2\pi t/T)$, T=12.5.

### 4.2. Results and Discussions of Experiments

**Figure 12:**
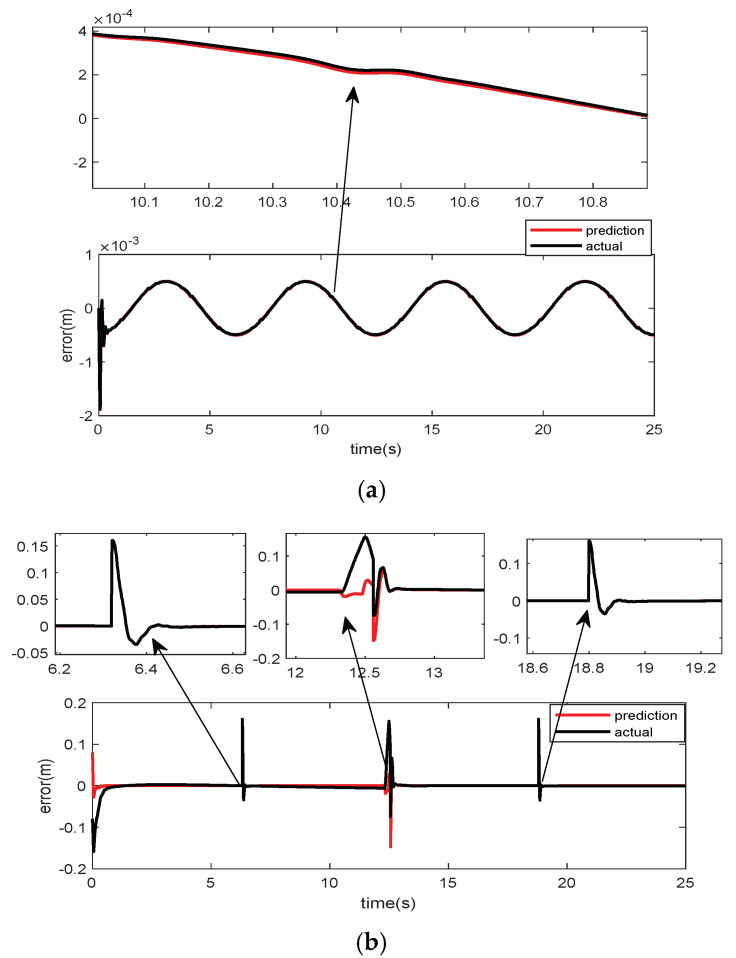
Comparison between predicted and actual errors: (**a**) comparison between actual and predicted error in *y*_1_; (**b**) comparison between actual and predicted error in *y*_2_.

Figure 12 shows the comparison between the prediction error and the actual error from the trained GRU network. According to (a) and (b) in Figure 12, the trained neural network almost precisely predicts the contouring errors for two completely different trajectories, demonstrating not only the efficiency but also the correctness of the prediction method. The accurate error predicted by the trained GRU network is used as feedforward compensation in the motion trajectories of the distributed coil magnetically levitated planar micromotor, thus constituting the GRU-ESO control strategy.

**Figure 13:**
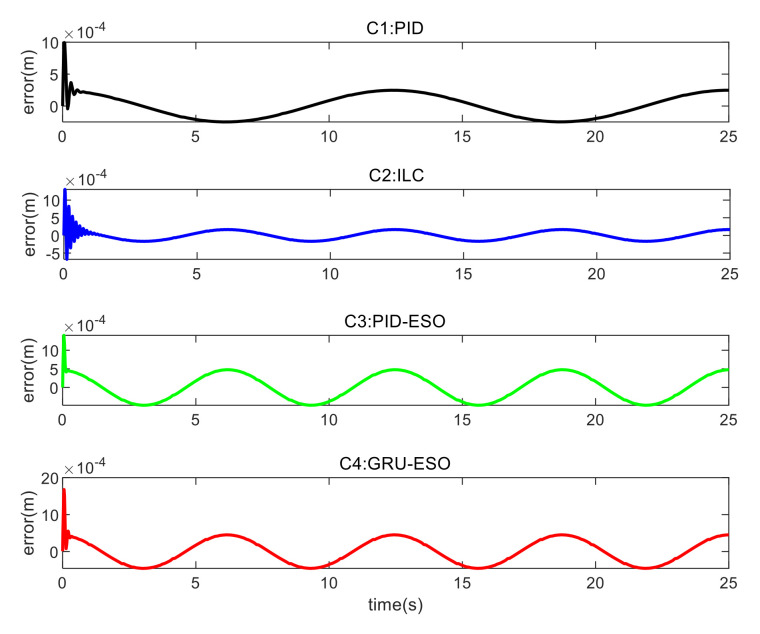
Tracking errors in Set 1 of *y*_1_.

**Figure 14:**
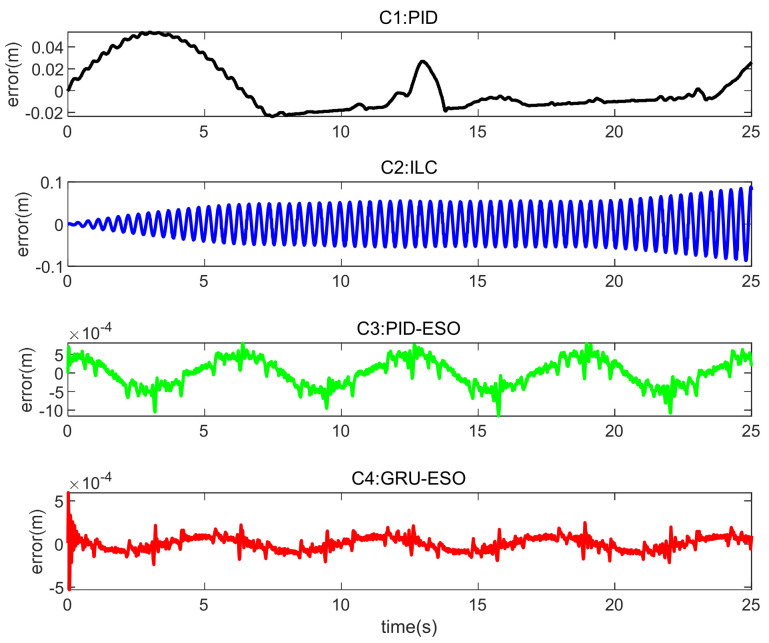
Tracking errors in Set 2 of *y*_1_.

**Figure 15:**
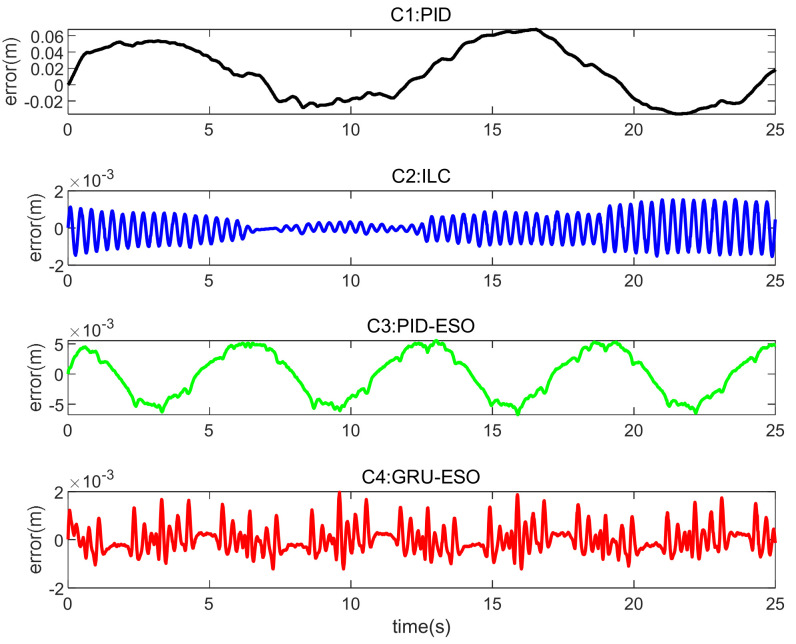
Tracking errors in Set 3 of *y*_1_.

**Figure 16:**
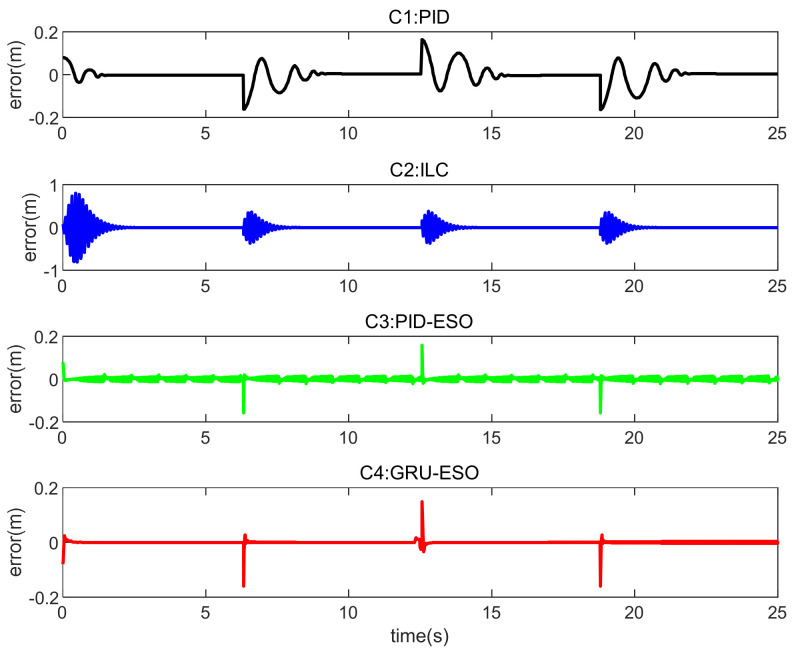
Tracking errors in Set 1 of *y*_2_.

**Figure 17:**
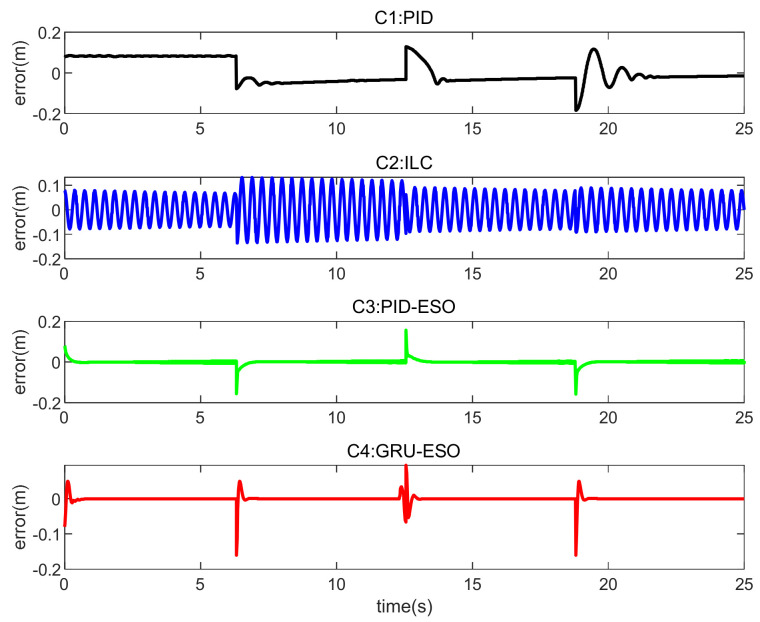
Tracking errors in Set 2 of *y*_2_.

**Figure 18:**
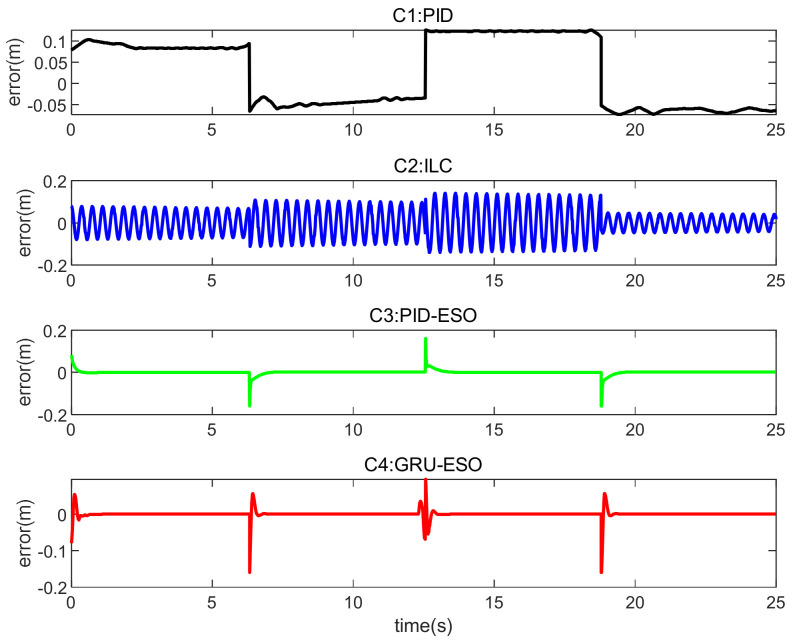
Tracking errors in Set 3 of *y*_2_.

The tracking errors for different controllers and sets in cases are shown in Figure 13, Figure 14 and Figure 15 and Figure 16, Figure 17 and Figure 18 respectively. The detailed quantitative indices are further shown in Table 3 and Table 4. Both Table 3 and Table 4 show that the proposed GRU-ESO control strategy has good tracking control accuracy.

In Figure 13, it can be observed that the tracking accuracy results for the different controllers in Set 1 are essentially similar. C1 (PID) performs slightly better, but still has significant residuals. The proposed C4 (GRU-ESO) further improves performance through feedforward compensation based on the GRU. It is worth noting that the performance of the proposed C4 is even comparable to that of C2 (ILC), while avoiding the time-consuming iteration steps in C2.

In Figure 14, in Set 2 with the random disturbances, the errors of controllers C1 and C2 are significant, indicating that these two controllers are susceptible to disturbances. The control performance of C3 (PID-ESO) is much better than that of C1 and C2. However, C4 still achieves better results, indicating that the proposed C4 control strategy is robust to disturbances.

In Figure 15, in Set 3 with the random disturbances and a payload of 1 kg, the result is similar to Set 2. The performances of C3 and C4 deteriorated to a large extent, because the appropriate reference modifications were changed from the nominal conditions. In contrast, C4 still maintains high accuracy. Therefore, the GRU-based feedforward compensation remains effective. The high precision of the proposed C4 under disturbances and parameter variations is the result of the intrinsic combination of GRU learning, adaptive control, and robust control.

In Figure 16, Figure 17 and Figure 18, it can be observed that the results obtained for Set 1, Set 2, and Set 3 in *y*_2_ are similar to those described above in *y*_1_. Figure 16, Figure 17 and Figure 18 show that the results are similar to those described above for *y*_2_. Set 1: In Figure 16, the tracking errors are mostly within the range of −0.2 m to 0.2 m. The proposed C4 exhibits small oscillations and quickly returns to a stable state when sudden changes occur. Set 2: In Figure 17, the proposed C4 shows the smallest tracking errors, indicating strong disturbance rejection capabilities. Set 3: In Figure 18, C1 and C2 are no longer suitable. The tracking error results for C3 and C4 are similar, but considering the error range, the proposed C4 demonstrates higher tracking control accuracy, indicating that the GRU-based feedforward compensation is effective.

In Table 3 and Table 4, it can be observed that under the reference trajectories *y*_1_ and *y*_2_, the control performances of C1 and C2 are relatively poor. The results for C3 and C4 are quite similar, but the proposed C4 has the smallest error and variance, indicating that GRU-based feedforward compensation has partially compensated for the errors and improved tracking control accuracy. The reference trajectory *y*_2_ is a square wave and still uses the same set of parameters in *y*_1_, so the overall tracking error in *y*_2_ is lower than that of *y*_1_. If the control parameters are modified, it may be improved overall. Nevertheless, the proposed C4 can still be obtained with a good control effect.

According to the experimental results, the proposed GRU-ESO achieves high control tracking accuracy. Additionally, it demonstrates robustness to external disturbances and model parameter variations. Compared to PID, ILC, and PID-ESO, which become worse in the presence of disturbances and parameter uncertainties, the proposed GRU-ESO still maintains satisfactorily high accuracy.

## 5. Conclusions

In this paper, a distributed coil magnetically levitated micromotion micromotor is designed for theoretical analysis and system modeling verification, and an improved ESO control strategy based on GRU neural network prediction is proposed. The control strategy uses ESO feedback control to provide robustness against external unknown disturbances; the GRU neural network is employed to accurately predict the system’s tracking error, with the predicted error used as a feedforward compensation to further improve tracking performance. Comparative experiments validate the proposed GRU-ESO for its excellent tracking performance and robustness to trajectory changes, model uncertainties, and disturbances.

## 6. Patents

The following patent is under examination: Multi-degree-of-freedom magnetic levitation motion platform and tracking control method (2024100782695).

## Figures and Tables

**Table 1:** Parameters of the mover permanent magnet.

	Length (mm)	Width (mm)	Height (mm)
**Big magnet**	10	10	5
**Small magnet**	10	5	2.5

**Table 2:** Parameters of the stator coil.

Internal Diameter (mm)	External Diameter (mm)	Height (mm)	Resistance Value (Ω)	Sentimental Value (mH)	Wire Diameter (mm)
8.1	4.5	14.5	9.9	7.5	0.25

**Table 3:** Tracking performance indices in *y*_1_.

Controller		C1: PID	C2: ILC	C3: PID-ESO	C4: GRU-ESO
Set1	$e_{RMS}$$(10^{-4}m$)	2.8431	3.3694	3.4176	3.2748
	$e_{M}$$(10^{-4}m$)	13.9491	13.033	13.999	12.779
Set2	$e_{RMS}$$(10^{-4}m$)	576.94	372.84	3.6067	0.79021
	$e_{M}$$(10^{-4}m$)	1840	877.6	11.568	5.9163
Set3	$e_{RMS}$$(10^{-4}m$)	353.25	6.6168	36.777	5.4125
	$e_{M}$$(10^{-4}m$)	676.58	15.32	67.459	19.725

**Table 4:** Tracking performance indices in *y*_2_.

Controller		C1: PID	C2: ILC	C3: PID-ESO	C4: GRU-ESO
Set1	$e_{RMS}$$(10^{-3}m$)	45.627	106.71	8.9061	7.2239
	$e_{M}$$(10^{-3}m$)	163.28	810.07	157.92	150.2
Set2	$e_{RMS}$$(10^{-3}m$)	57.694	66.821	9.9763	9.609
	$e_{M}$$(10^{-3}m$)	184	135.3	157.29	150.99
Set3	$e_{RMS}$$(10^{-3}m$)	84.88	67.89	12.176	11.078
	$e_{M}$$(10^{-3}m$)	125.63	141.22	160.56	151.98

## Data Availability

All data for this study have been experimentally generated and have been included in this paper.

## Appendix A

In the permanent magnet array, the magnetic induction strength generated by magnetic steel with six different magnetization directions is denoted, respectively, as ${B_{x}}^{\prime }$, ${B_{y}}^{\prime }$, ${B_{z}}^{\prime }$,${B_{-x}}^{\prime }$, ${B_{-y}}^{\prime }$, ${B_{-z}}^{\prime }$:

(A1) $$\begin{array}{ll} {B_{z}}^{\prime } & =({B_{zx}}^{\prime },{B_{zy}}^{\prime },{B_{zz}}^{\prime })\\ & =\sum_{i=0}^{3}\sum_{j=0}^{3}\frac{1+{\left(-1\right)}^{i+j}}{2}f[x-(a_{b}+h_{s})i,y-(b_{b}+h_{s})j,z,J_{1}] \end{array},$$

(A2) $$\begin{array}{ll} {B_{-z}}^{\prime } & =({B_{-zx}}^{\prime },{B_{-zy}}^{\prime },{B_{-zz}}^{\prime })\\ & =\sum_{i=0}^{3}\sum_{j=0}^{3}\frac{1-{\left(-1\right)}^{i+j}}{2}f[-x+a_{b}+(a_{b}+h_{s})i,y-(b_{b}+h_{s})j,-z+h_{b},J_{1}] \end{array},$$

(A3) $$\begin{array}{ll} {B_{y}}^{\prime } & =({B_{yx}}^{\prime },{B_{yy}}^{\prime },{B_{yz}}^{\prime })\\ & =\sum_{i=0}^{4}\sum_{j=0}^{3}\frac{1+{\left(-1\right)}^{i+j}}{2}f[-x+a_{b}+(a_{b}+h_{s})j,z,y+h_{s}+(b_{b}+h_{s})i,J_{2}] \end{array},$$

(A4) $$\begin{array}{ll} {B_{-y}}^{\prime } & =({B_{-yx}}^{\prime },{B_{-yy}}^{\prime },{B_{-yz}}^{\prime })\\ & =\sum_{i=0}^{4}\sum_{j=0}^{3}\frac{1+{\left(-1\right)}^{i+j}}{2}f[-x+a_{b}+(a_{b}+h_{s})j,-z+h_{b},-y+(b_{b}+h_{s})i,J_{2}] \end{array},$$

(A5) $$\begin{array}{ll} {B_{x}}^{\prime } & =({B_{xx}}^{\prime },{B_{xy}}^{\prime },{B_{xz}}^{\prime })\\ & =\sum_{i=0}^{4}\sum_{j=0}^{3}\frac{1-{\left(-1\right)}^{i+j}}{2}f[y-(a_{s}+h_{s})j,z,x+h_{s}-(a_{b}+h_{s})i,J_{2}] \end{array},$$

(A6) $$\begin{array}{ll} {B_{-x}}^{\prime } & =({B_{-xx}}^{\prime },{B_{-xy}}^{\prime },{B_{-xz}}^{\prime })\\ & =\sum_{i=0}^{4}\sum_{j=0}^{3}\frac{1+{\left(-1\right)}^{i+j}}{2}f[y-j(b_{b}+h_{s})j,-z+h_{b},-x+(a_{b}+h_{s})i,J_{2}] \end{array},$$

where i=0~3 and j=0~3 are the (i+1) and (j+1) magnets along the *x*-axis and *y*-axis directions, respectively; $a_{b}$, $b_{b}$, and $h_{b}$ are the length, width, and height of the big magnet, respectively; and $a_{s}$, $b_{s}$, and $h_{s}$ are the length, width, and height of the small magnet, respectively.
